# Compensatory neuromuscular junction adaptations of forelimb muscles in focal cortical ischemia in rats

**DOI:** 10.1002/brb3.1472

**Published:** 2020-01-31

**Authors:** Yisel Carolina Estrada‐Bonilla, Paula Aiello Castro de Souza‐Tomé, Fernanda María Faturi, Rafaella Mendes‐Zambetta, Anna Carolyna Lepesteur‐Gianlorenço, Gabrielle Croti, Theresa A. Jones, Thiago Luiz Russo

**Affiliations:** ^1^ Physiotherapy Deparment – (DFisio) Laboratorio de Pesquisa em Fisioterapia Neurológica – LaFiN Federal University of São Carlos São Carlos Brazil; ^2^ Body, Subjetct and Education Research Group Physical Culture, Sports and Recreation Saint Thomas University Bogotá DC Colombia; ^3^ Department of Psychology Institute for Neuroscience University of Texas at Austin Austin TX USA

**Keywords:** cerebral ischemia, induced stroke, motor endplate, motor performance, neuromuscular junction, reaching task

## Abstract

**Introduction:**

Upper limb movements are affected frequently by brain ischemia (BI). Mechanisms involved in recovery and compensatory movements have developed several studies. However, less attention is given to skeletal muscles, where neuromuscular junction (NMJ) has an important role on muscle tropism and functional performance.

**Methods:**

Animals were divided into two groups: control (C) and BI. Then, animals were skilled to perform single‐pellet retrieval task, following these procedures: habituation, shaping, and single‐pellet retrieval task. BI was induced using stereotaxic surgery in order to apply endothelin‐1 in motor cortex, representative of movements of dominant paw. Reaching task performance was evaluated by single‐pellet retrieval task 1 day before BI induction, 4 and 15 days after BI induction. After that, biceps, triceps, fingers flexor, and extensor muscles were extracted. NMJ was assessed in morphometric characteristics (total area, total perimeter, and feret). Muscle fiber cross‐sectional area and connective tissue percentage were also evaluated for characterization. Student's *t* test was used for comparisons between C and BI groups. Tau Kendall's correlation was applied among variables from BI group.

**Results:**

An increase in all NMJ morphometric parameters, as well as increase of atrophy and fibrosis in BI group compared with C. There was a high level of direct correlation between mean values of NMJ morphometry with percentage of success in reaching task in BI group.

**Conclusion:**

Brain ischemia‐induced NMJ compensatory expansion, muscle atrophy, and fibrosis in forelimb muscles that are related to reaching performance.

## Graphical abstract

Reaching task performance. Brain ischemia and his effects in Reaching task.

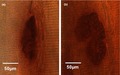

## 1 INTRODUCTION

Stroke is considered the second cause of death and the first cause of disability worldwide (World Health Organization – WHO, 2016). More than half of individuals with stroke present impaired function of upper limbs, affecting the independence in activities of daily living (Jones, 2017). Upper limb deficits observed in poststroke subjects may be related to sensorimotor control disruption that includes, for example, impairments in strength (Avila, Romaguera, Oliveira, Camargo, & Salvini, 2013; Santos et al., 2016; Turner, Tang, Winterbotham, & Kmetova, 2012). Muscle weakness has been associated with compensatory strategies of movement due to the lesion of the motor cortex and its descending projection pathways, reduced muscle activation and incoordination (Hatem et al., 2016; Li, Tong, & Hu, 2007; Niessen et al., 2008), and anatomic modifications on skeletal muscle, such as muscle fiber phenotype shift, fibrosis, and atrophy (Klein, Brooks, Richardson, McIlroy, & Bayley, 2010; Li et al., 2007; Lieber & Fridén, 2004; Raghavan, 2015).

The neuromuscular junction (NMJ), or motor endplate, has a keyhole in the regulation of contractile activity of skeletal muscles, because it transmits the action potentials from motor neurons to muscle fibers. Changes in NMJ morphology, such as the size of the nerve terminal and number of active zones, were described in different diseases (Tinginac, Brenner, & Ruegg, 2015). However, there is no available information in the literature about how stroke can affect NMJ plasticity.

Considering that the brain lesion and subsequent interruption of the upper motoneuron pathways caused reduction on the number of motor units in the paretic upper limbs and the motor recovery in poststroke is associated with crossing of corticospinal tract (CST) fibers from the lesion‐spared, intact motor cortex to the stroke‐denervated side of the spinal cord (Keisure et al., 2006; Tinginac et al., 2015). It is reasonable to assume that NMJ can be altered due to stroke. Furthermore, it was demonstrated compensatory mechanisms of NMJ to maintain function and performance during aging, such as the increase in the length of branching and in the overall area of the postsynaptic membrane (Walh et al., 2014).

There are remarkable homologies in the movement patterns used by rodents and humans to perform reach to grasp tasks. There is also resemblance in the impairments and compensatory movement patterns that result from unilateral motor system damage showing that focal ischemic models are important for translational studies (Jones, 2017). Therefore, the aim of this study was to describe the morphometric modifications of the NMJ, muscle cross‐sectional area, and proliferation of connective tissue in muscles that are involved in very coordinated movements of forelimb, as reaching and grasping task, in focal cortical ischemic model in rat, as well as the association of such morphometric changes with the motor performance. The hypotheses raised in this study are that brain ischemia might generate muscle atrophy, fibrosis, and expansion of the NMJ. In addition, these changes might be associated with reaching and grasping success.

## 2 METHODS

The study was conducted according to the international standard of animal experimentation after the approval by the Ethics Committee on the use of animals (ECUA) of the Federal University of Sao Carlos (UFSCar). The experiment followed recommendations from ARRIVE guidelines, and it is in accordance with the National Institutes of Health guide for the care and use of animal laboratories.

### 2.1 Animals and experimental design

Twelve 3‐month‐old male Wistar rats were pair housed in cages in the Department of Physical Therapy biotereo at the Federal University of São Carlos (UFSCar). A 12/12‐hr light/dark cycle was performed with water access ad libitum. Animals were daily handled 2–3 weeks prior to the experiment, and all behavioral procedures were performed in the same room. Prior to the start of behavioral methods, animals were placed on scheduled feeding of 15 g of rat chow given once per day (to ensure rats will not sit at the time of training). Weights were monitored throughout the study.

Animals were submitted to the training chamber during 2 consecutive days (habituation period), then more 2–8 days of procedures of shaping on the single‐pellet retrieval task to determine forelimb preference (dominance). After that, animals were trained in reaching task for 10 days. After training, at −1 day (pre‐BI Induction), animals underwent measurement of forelimb functional test (single reaching task test) and submitted to the Cerebral Ischemia induction. In animals that not were part of the Brain Ischemia group (BI), process of Brain Ischemia sham induction was performed (with stereotaxic surgery), but without endothelin 1 application. Functional test (single reaching task test) was performed for each of the animals in two more moments: 3 days after brain ischemia/sham induction, 4 days post‐BI induction (final phase of acute BI), and 1 day before euthanasia (corresponding to 15 days of cerebral ischemia/sham induction).

Groups were defined as follow:

1. Brain ischemia group (BI Group; *n* = 6): animals submitted to all procedures, including habituation period, shaping and training period, and cerebral ischemia induction.
2. Control group (C group; *n* = 6): animals submitted to the procedures of habituation, shaping period, and training period. These animals went through sham of cerebral ischemia induction. All the animals were euthanized 15 days after performing the second functional forelimb test.

#### 2.1.1 Habituation and shaping

All animals were submitted to a habituation period (20 min/day) for 2 consecutive days in a Plexiglas reaching chamber (30 cm long by 35 cm high by 15 cm wide) with a tall narrow window (1 cm wide and 3 cm high) in the center of the 15‐cm‐wide wall. For shaping, animals were placed in the same reaching chamber for 20 min. Animals reached with a forelimb through a small window for Froot loops (Kellogg's), which were placed in front of a block, approximately 3 cm in height. The wells were centered with the left and right edges of the window at a distance of 1 cm from the window. A small Plexiglas rod approximately 2 mm in diameter adhered to the base of the reaching window created a barrier that prevented animals from scraping the pellets into the chamber and also reduced attempts to use the tongue to retrieve pellets. When 20 consecutive reach attempts were performed with one limb during a 20‐min session, this limb was identified as the preferred limb. Once animals reach these criteria, preoperative shaping was ceased and the reach training before surgery started.

#### 2.1.2 Training on the single reaching task

Training on the single‐pellet retrieval task was carried out in the reaching chamber. During training, a Plexiglas wall was inserted into the reaching chamber ipsilateral to the animal's trained limb and pellets were placed so that the animal could catch the pellet with the dominant front leg. This wall effectively forces the animals to use the forelimb chosen by the experimenter for the reaching task. In the initial design of the apparatus, the inner chamber wall was placed at a distance of 1.5 cm from the reaching window.

The training with the preferred limb was performed in all groups 10 days before the assessment of forelimb asymmetry and reach performance by single‐pellet retrieval tests. Animals were trained for 30 trials or a cutoff time (20 min), which ever come first. A reaching trial consisted of the animal either successfully grabbing the pellet and bringing it directly to its mouth (success), dropping the pellet before bringing it to its mouth, failing to grasp the pellet after five reaches, or knocking the pellet out of its well. At the end of each reaching trial, a pellet was dropped into either the front or the back of the reaching chamber to “reset” the animals and so that a new pellet was placed into its appropriate well. After induction/sham of cerebral ischemia was performed, no further training of reaching task was performed.

#### 2.1.3 Testing on the reaching task

Testing on the single‐pellet retrieval task was performed on day −1 (before sham/BI induction), 3 days after sham/BI induction, 4 days after BI induction (final of acute phase of BI), and 14 after performing the third functional forelimb test. Reaching performance was calculated by dividing the total number of successful reaches by the total number of reach attempts with preferred limb [(total success/total reach attempts) × 100], which corresponds to percent successful reaches. Test was performed during 20 min (Adkins et al., 2015).

### 2.2 Surgery—BI induction

A focal unilateral lesion of the forelimb representation area of the sensorimotor cortex (SMC) contralateral to the preferred limb was created. Animals were anesthetized using intraperitoneal (i.p.) injections of xylazine (12 mg/kg) and ketamine (95 mg/kg), and placed in a stereotaxic apparatus. A midsagittal incision and a craniotomy were made between 2.5 mm anterior, 0.5 mm posterior, and 3.0–4.5 mm lateral to Bregma. Pia mater was exposed by removal of durra in the area underlying the craniotomy. Endothelin‐1 (ET‐1, 80 μM, 0.2 μg/μl; 8 μl total volume administered), a vasospasm‐inducing peptide, was topically administered with a 10 µl volume pipette for 10 min before the skin was sutured. During all surgery, animal's temperature was controlled using a heat pad. Rats were allowed 4 days of recovery before postoperative behavioral manipulation starts (training; Adkins, Voorhies, & Jones, 2004).

### 2.3 Muscle sample collection

Biceps (B), triceps (T), fingers flexor (FF), and fingers extensor (FE) muscles were isolated, removed and weighed. Each muscle was divided into two parts. The proximal fragment was immersed in glutaraldehyde solution and then was used for the nonspecific esterase technique. Distal fragment of each muscle was used for histological morphometry, precooled in liquid nitrogen and stored at −80°C, and then, used to measure both the cross‐sectional area (CSA) of the muscle fibers and % of connective tissue.

### 2.4 Brain morphology

The brain was removed and placed in paraformaldehyde fixative solution for 24 hr and was then placed in a sucrose solution and kept refrigerated. Histological cross sections (30 µm) from the motor cortex area injured were made and stained with a Nissl stain (cresyl violet) and were used to confirm the injury. Only animals that presented focal cortical damage in morphology were included in the analysis. Each of the sheets that were obtained after applying Nissl technique were scanned using 3d histech panoramic desk equipment, using panoramic viewer software (3d histech) to edit the images obtained.

### 2.5 Muscle fiber CSA and percentage of connective tissue

Histologic serial cross sections were obtained from the biceps, triceps, fingers flexor, and fingers extensor in a cryostat microtome (Leica; CM1860). A histologic cross section (10 µm) stained with toluidine blue was selected to measure the CSA under a light microscope (Axiovision 3.0.6 SP4; Carl Zeiss) using morphometric analysis (ImageJ software, version 1.43u; National Institutes of Health). The CSA of each muscle was obtained by measuring 100 fibers located in the central region of the section. The % of connective tissue in each muscle was evaluated using ImageJ software analysis, making a grid on an photograph of each muscle, to then count the number of intersections of the grid that fell into the connective tissue, to proceed to calculate the percentage of points within the conjunctive period versus the number of points that the traced grid had (number of points or intersections within connective tissue/points or intersections of quadricula × 100).

### 2.6 NMJ analysis—nonspecific esterase technique

The surface portions of biceps, triceps, fingers flexor, and fingers extensor were trimmed to the NMJ portion (containing the motor point), which was cut lengthwise into three or four slices. The resulting material was subjected to the nonspecific esterase technique (Lehrer technique) to characterize the NMJ.

### 2.7 Morphometric analysis

Total area, total perimeter, and feret were measured on 50 junctions with a light microscope (Axiovision 3.0.6 SP4; Carl Zeiss). Relative planar area of each motor plate was also calculated taking into account the ratio between total area/major diameter previously evaluated. The measures were analyzed with ImageJ software (Image J version 1.43u; National Institutes of Health; Adkins et al., 2004; Allred, Maldonado, & Jones, 2005).

### 2.8 Statistics

All variables (total body weight, reaching performance—% of success, NMJ morphometry and muscle morphometry—CSA and % of connective tissue), showed normality and homogeneity distribution according to Shapiro–Wilk e Levene tests, respectively. For variables of total body weight, morphometry of NMJ, and histological morphometry of muscle tissue, comparison of means was applied by Student's *t* test, comparing pre‐BI/sham moment and 15 days post‐BI induction (after 15d), within and between groups. For variable of motor performance (% of success), comparison of means was made by Student's *t* test, comparing moments of performance of the range test −1d (1 day pre‐BI induction) and 3 days of BI induction (after 3d BI) and this last moment compared too with 15 days after BI moment (15d after BI), within, and between groups too. Likewise, two types of correlations were established (with Tau Kendall correlation coefficient): (a) between NMJ mean area, perimeter, and feret in each muscle evaluated—individuals in the BI group, with motor performance (% success) obtained in the range test performed after 15 days post‐BI induction (after 15d BI) and (b) between NMJ mean area, perimeter, and feret in each evaluated muscle—individuals of the BI group with histological morphometric characteristics evaluated (CSA and % of connective tissue) of each muscle evaluated in individuals of the BI group. Magnitude of correlations was based on the Munro's classification (low [0.26–0.49], moderate [0.50–0.69], high [0.70–0.89], and very high [0.90–1.00]; Munro, 2001).

## 3 RESULTS

### 3.1 Total body weight

**Figure 1:**
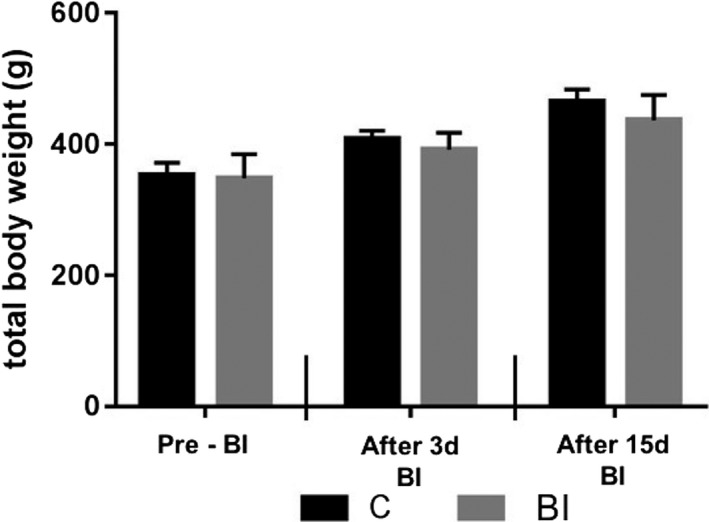
Mean values of total body weight in animals from control group (C) and brain ischemia group (BI). No differences were found in weight of animals of the BI group compared with weight of animals of C group. Data are expressed as mean ± *SEM*. Number of animals: six per group

The C and BI groups presented similar values of total body weight comparing first and second moment (pre‐BI) with second moment of assessment (3d after BI; *p* = .201) and comparing second moment (3d after BI) with third moment of assessment (15d after BI; *p* = .198; Please see Figure 1).

### 3.2 Focal cortical damage

**Figure 2:**
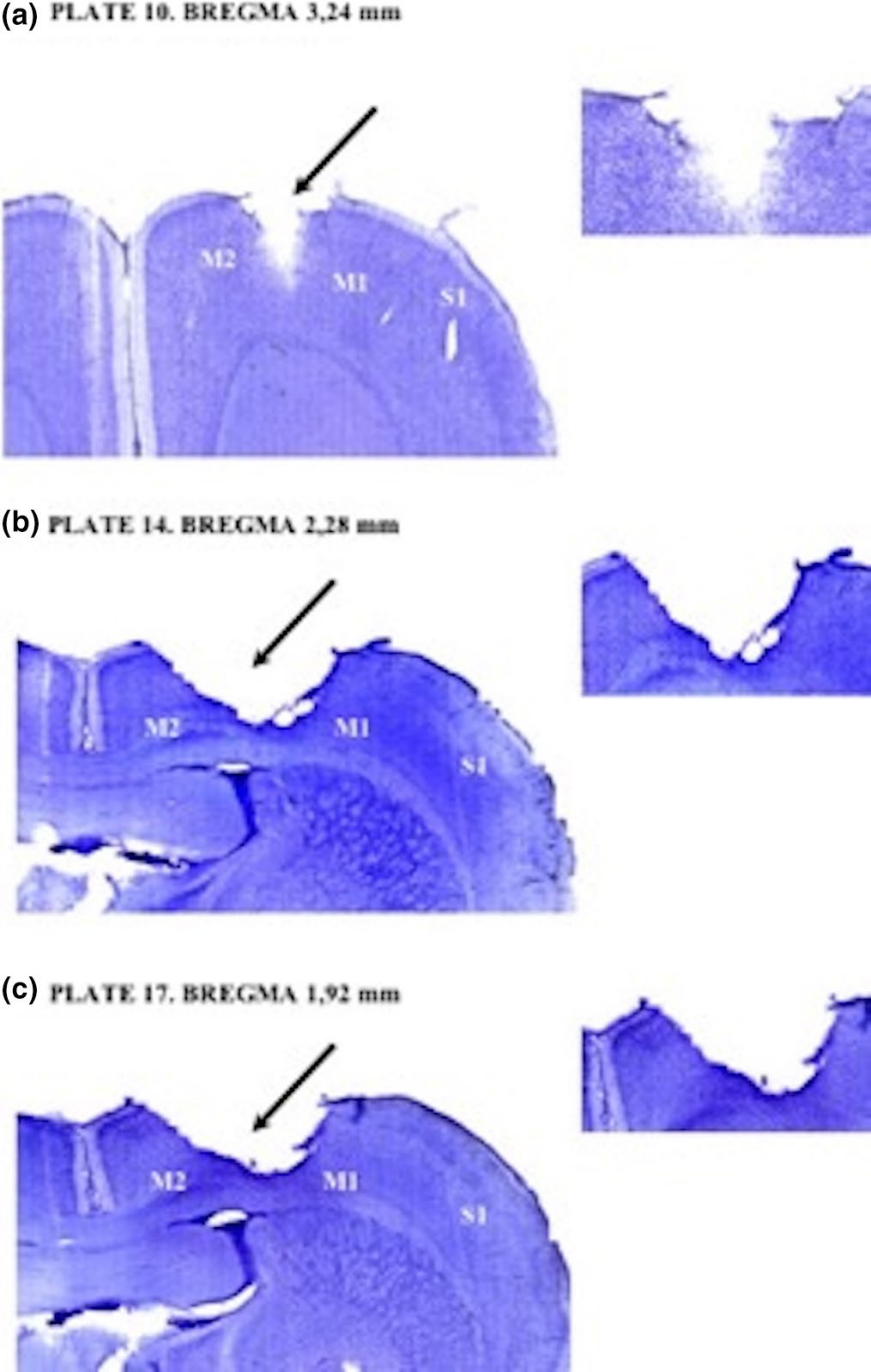
Brain ischemia injury in motor cortex: motor cortex area 1 (M1), motor cortex area 2 (M2), supplementary motor cortex area (S1). In (a): Plate 10 (Bregma 3.24 mm); In (b): Plate 14 (Bregma 2.28 mm). In (c): Plate 17 (Bregma 1.92 mm)

**Figure 3:**
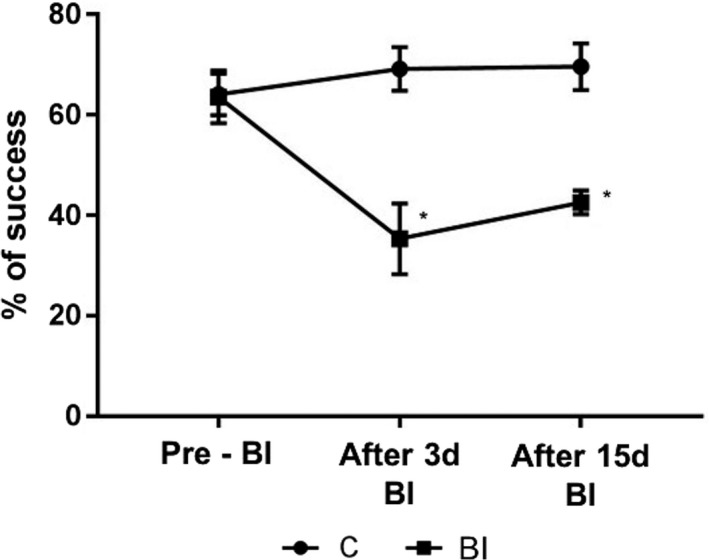
Percentage of success in motor performance. *p < .001 when brain ischemia (BI) is compared to C. Control (C) and BI groups; Pre‐BI, the moment before BI induction. After 3d induction (final acute phase of BI), and After 15d BI (chronical phase of BI). Data are expressed as mean ± *SEM*. Number of animals: six per group

Animals of BI group were submitted to ET‐1 brain ischemia presented cortical damage in brain morphology (Figure 3). Endothelin‐1 produced reliable focal infarcts with neuronal death on primary (M1) and secondary (M2) motor cortices, and primary somatosensory cortex. Animals of C group were submitted only to stereotaxic surgery without ET‐1 application (See Figure 2).

### 3.3 Motor performance

**Figure 4:**
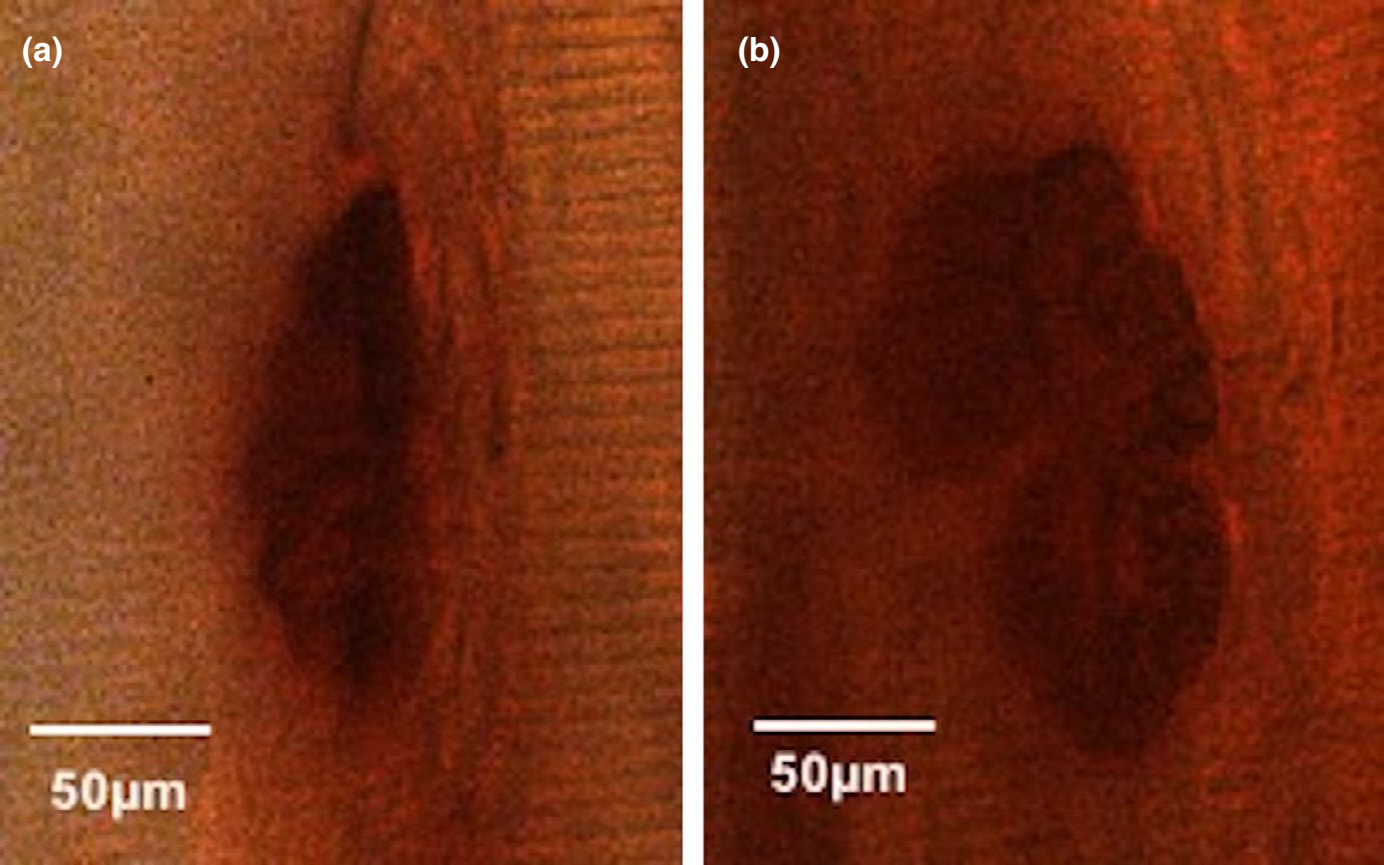
Representative morphology of neuromuscular junction stained with nonspecific esterase technique from biceps muscles. (a) control group; (b) cerebral ischemia group. Scale bar: 50 µm

At the pre‐BI induction moment, both BI and C groups presented similar percentage of reaching success (*p* = .201; Figure 4). However, 3 days after BI induction, BI group decreased its percentage of success compared with control values (*p* = .0001; Figure 3). Likewise, when comparing the percentage of success obtained by the BI group in the third moment of evaluation of reaching task (15d after BI), although a slight improvement is observed in the same individuals, a percentage of event decreased even compared with the control group (*p* = .0001; Figure 4). Making a brief description of qualitative characteristics of reaching task (in the individuals of the group BI), it is observed especially in the third evaluation moment, compensations such as trying to reach the pellet with the other forelimb (no dominant forelimb), as well as bringing the head and mouth to the training box window, to try to reach the pellet. Reaching movement exhibited in BI group evidences dysmetria and bradykinesia.

### 3.4 NMJ morphology

Rats from BI group present clear morphologic signals of increased NMJs' expansion, compared with C. Furthermore, NMJs presented more robust and larger form, with a loss of typical “pretzel” shape (compared with NMJs in the C group; Figure 3). Morphometric analyses showed an increase in NMJ total area, perimeter, feret, and relative planar area are in all evaluated muscles when compared to control (Table 1).

**Table 1:** Morphometric analysis of NMJ, normalized muscle weight, muscle fiber cross‐sectional area muscle fiber, and percentage of connective tissue from biceps, triceps, fingers flexor, and fingers extensor muscles from brain ischemia group (BI) and control group (C)

Morphometric parameters	Control (C)	Cerebral ischemia (CI)	*p*
Biceps			
NMJ mean area (µm^2^)	243.7 ± 81.7	439.5 ± 37.4***	.0001
NMJ mean perimeter (μm)	65.9 ± 10.5	92 ± 9.5***	.0001
NMJ mean feret (μm)	24.9 ± 37.6	25 ± 9.2	.992
NMJ mean relative planar area (area/major perimeter)	8.8 ± 1.7	13.0 ± 1.7*	.05
Normalized muscle weight (%)	0.0007 ± 0.0001	0.0007 ± 0.0004	.713
Muscle fiber CSA (μm^2^)	199.6 ± 18.9	122.7 ± 11.9***	.0001
Connective tissue (%)	6.3 ± 0.6	7.2 ± 0.5	.330
Triceps			
NMJ mean area (µm^2^)	275.5 ± 96.8	437 ± 39.3***	.0001
NMJ mean perimeter (μm)	70.8 ± 13.4	141 ± 0.8***	.0001
NMJ mean feret (μm)	27.4 ± 5.1	51.5 ± 7.5*	.01
NMJ mean relative planar area (area/major perimeter)	9.04 ± 1.6	11.3 ± 1.3	.14
Normalized muscle weight (%)	0.003 ± 0.0005	0.003 ± 0.003	.659
Muscle fiber CSA (μm^2^)	253.6 ± 23.6	208.6 ± 20.6***	.0001
Connective tissue (%)	5.3 ± 0.5	6.3 ± 0.8	.304
Fingers flexor			
NMJ mean area (µm^2^)	217.9 ± 78.1	443 ± 65.2***	.0001
NMJ mean perimeter (μm)	62.7 ± 12.2	64.6 ± 9.9	.856
NMJ mean feret (μm)	24.4 ± 4.9	24.3 ± 3.3	.962
NMJ mean relative planar area (area/major perimeter)	7.9 ± 1.3	8.3 ± 1.2	.730
Normalized muscle weight (%)	0.0015 ± 0.0001	0.0013 ± 0.0005**	.01
Muscle fiber CSA (μm^2^)	70.3 ± 7.0	52.3 ± 2.3***	.0001
Connective tissue (%)	5.7 ± 0.9	13 ± 0.6**	.01
Fingers extensor			
NMJ mean area (µm^2^)	205.1 ± 68.8	551.2 ± 45.3***	.0001
NMJ mean perimeter (μm)	59.9 ± 10.1	75.6 ± 16.9*	.04
NMJ mean feret (μm)	22.8 ± 3.8	28.1 ± 2.6	.07
NMJ mean relative planar area (area/major perimeter)	8.6 ± 1.3	11.2 ± 1.8**	.01
Normalized muscle weight (%)	0.0005 ± 0.0002	0.0004 ± 0.0002	.212
Muscle fiber CSA (μm^2^)	84.1 ± 5.2	76.3 ± 5.3*	.01
Connective tissue (%)	5.3 ± 0.5	9.5 ± 1.0*	.02

Note

Data are expressed as mean ± *SEM*. Number of animals: six per group.

Abbreviations: CSA, cross‐sectional area; NMJ, neuromuscular junction.

* *p* < .05–*p* < .01, **p < .0001, when BI is compared with C and ***p < .0001, when CI is compared to C.

### 3.5 Muscle weight, muscle fiber cross‐sectional area, and percentage of connective tissue

Finger flexors muscle weight reduced its values in BI compared with C (Table 1). Reduction in muscle fiber CSA and increased percentage of connective tissue were observed in all muscles of BI compared with C (Table 1).

### 3.6 Correlation between morphometric NMJ parameters and motor performance in BI group

A high positive correlation was observed for NMJ mean area, perimeter, or ferret with percentage of success during reaching task for all muscles evaluated (Table 2), for example, as bigger the area, perimeter, or ferret, as better the motor performance.

**Table 2:** Correlations between: (a) morphometric characteristics of NMJ (area, perimeter, and feret) and motor performance in brain ischemia (BI) group 15 days after induction and between: (b) morphometric characteristics of NMJ (area, perimeter, and feret) and morphometric characteristics of measured muscles (muscle fiber cross‐sectional area and percentage of connective tissue) in BI group

Correlated variables	Tau Kendall correlation level (T)	T
Biceps		
NMJ area of biceps versus % of success in motor reach task test	.838	.037*
NMJ perimeter of biceps versus % of success in motor reach task test	.838	.037*
NMJ ferret of biceps versus % of success in motor reach task test	.856	.030*
NMJ area of biceps versus CSA of biceps	−.971	.0001**
NMJ perimeter of biceps versus CSA of biceps	−.972	.0001**
NMJ ferret of biceps versus CSA of biceps	−.911	.01*
NMJ area of biceps versus % of connective tissue of biceps	.854	.03*
NMJ perimeter of biceps versus % of connective tissue of biceps	.855	.03*
NMJ ferret of biceps versus % of connective tissue of biceps	.980	.0001**
Triceps		
NMJ area of triceps versus % of success in motor reach task test	.838	.037*
NMJ perimeter of triceps versus % of success in motor reach task test	.799	.057*
NMJ ferret of triceps versus % of success in motor reach task test	.846	.034*
NMJ area of triceps versus CSA of triceps	−.921	.01*
NMJ perimeter of triceps versus CSA of triceps	−.993	.0001**
NMJ feret of triceps versus CSA of triceps	−.992	.0001**
NMJ area of triceps versus % of connective tissue of triceps	.898	.01*
NMJ perimeter of triceps versus % of connective tissue of triceps	.841	.03*
NMJ feret of triceps versus % of connective tissue of triceps	.837	.03*
Fingers flexor		
NMJ area of fingers flexor versus % of success in motor reach task test	.838	.037*
NMJ perimeter of fingers flexor versus % of success in motor reach task test	.837	.037*
NMJ ferret of fingers flexor versus % of success in motor reach task test	.839	.037*
NMJ area of fingers flexor versus CSA of fingers flexor	−.728	.101*
NMJ perimeter of fingers flexor versus CSA of fingers flexor	−.729	.101*
NMJ feret of Fingers flexor versus CSA of Fingers flexor	−.729	.100*
NMJ area of fingers flexor versus % of connective tissue of fingers flexor	.894	.01*
NMJ perimeter of fingers flexor versus % of connective tissue of fingers flexor	.895	.01*
NMJ feret of fingers flexor versus % of connective tissue of fingers flexor	.895	.01*
Fingers extensor		
NMJ area of fingers extensor versus % of success in motor reach task test	.837	.037*
NMJ perimeter of fingers extensor versus % of success in motor reach task test	.838	.037*
NMJ ferret of fingers extensor versus % of success in motor reach task test	.838	.037*
NMJ area of fingers extensor versus CSA of fingers extensor	−.999	.0001**
NMJ perimeter of fingers extensor versus CSA fingers extensor	−.999	.0001**
NMJ feret of fingers extensor versus CSA fingers extensor	−.998	.0001**
NMJ area of fingers extensor versus % of connective tissue of fingers extensor	.867	.02*
NMJ perimeter of fingers extensor versus % of connective tissue of fingers extensor	.867	.02*
NMJ feret of fingers extensor versus % of connective tissue of fingers extensor	.866	.02*

Note

Data are expressed as mean ± SEM. Number of animals: six per group.

Abbreviations: CSA, cross‐sectional area; NMJ, neuromuscular junction.

* *p* < .05–*p* < .01 and **p < .0001, when CI is compared to C.

### 3.7 Correlation between morphometric NMJ parameters and muscle morphometric parameters

Moderate to high inverse correlations were observed for NMJ mean area, perimeter, and ferret versus muscle fiber CSA in all evaluated muscles. Moderate to high positive correlations were observed for NMJ mean area, perimeter, and ferret versus percentage of connective tissue in each evaluated muscle (Table 2).

## 4 DISCUSSION

One important issue observed in this study was the expansion of NMJ of forelimb muscles after focal cortical ischemia induced by endothelin‐1. Interesting, NMJ total area, perimeter, and ferret presented positive correlations in reaching and grasping performance. These results provided insights about neuromuscular compensatory mechanisms for ensuring forelimb performance during coordinated activities.

It was described that agrin, an important glycoprotein for ensuring the stability, number, and grouping of acetylcholine receptors (AChR) in the postsynaptic membrane, is increased in the acute and subacute phase after cerebral ischemia (Carin‐Levy et al., 2006; Schebakov et al., 2016; Scherbakov & Doehner, 2011). The increase in agrin was interpreted as a compensatory mechanism of the central nervous system (CNS), due to reductions in CNS inputs, to ensure muscle activity. However, this attempt to compensate disturbs of CNS is insufficient. For example, the proportion of activated ACRs were not distributed regularly in the active crests of the neuromuscular junction, with gaps of nonactive areas (Engel, 2004). This irregularity in the AchR's distribution would generate loss of neuronal drive efficiency; in other words, despite having more AChRs anchored in the postsynaptic membrane, many of them are dysfunctional (Carin‐Levy et al., 2006; Scherbakov & Doehner, 2011).

Furthermore, increase of AChRs in active and nonactive crests was associated with expansion of the NMJ (Carin‐Levy et al., 2006; Engel, 2004). Nevertheless, this larger NMJ lost its typical “pretzel” evaginations (Engel, 2004). Such modifications observed in poststroke models are very similar to those from aging models (for review see Tintgnac). Therefore, considering the increase in NMJ and the correlations with functional status observed in this study, it is possible to suppose that similar compensatory mechanisms are happening.

Another important result observed in this study is related to the muscle atrophy and increase in connective tissue proliferation in forelimb muscles due to focal cortical ischemia. This is the first study reporting such skeletal muscle alterations in forelimb in the late‐term of brain ischemia due to endothelin‐1. In addition, the increase in muscle fiber atrophy and fibrosis was associated with worse functional performance in the single‐pellet retrieval test. A recent study described muscle atrophy in the hind limb 3 days after cerebral ischemia (60 min of the middle cerebral artery occlusion, MCAO) in the paretic limb in mice and severe sensorimotor deficits during walking (Chleboun, France, Crill, Braddock, & Howell, 2001). Paretic hind limb muscle atrophy was also observed using the same model of brain ischemia at 7 days postinjury (Thompson, 2002). The reduced inputs from the CST can be considered the trigger of muscle atrophy.

More recently, the concept of stroke‐related sarcopenia was created to differentiate from sarcopenia observed in aging. In resume, there is a rapid decline of muscle mass with structural alterations (atrophy, fibrosis, muscle shift toward fast‐twitch fibers), and functional deficits due to brain lesion (Schebakov et al., 2016). Skeletal muscle atrophy occurs as a result of imbalance between the anabolic and catabolic processes and the main pathway of myoproteins' degradation is the ubiquitin‐proteasome pathway (Bonaldo & Sandri, 2013; Sandri, 2008). Anabolic pathways and neurotrophic factors seem to be compromised too. Both insulin‐like growth factor 1 (IGF‐1) and brain‐derived neurotrophic factor (BDNF) serum levels are decreased in chronic poststroke subjects (Santos et al., 2016; Silva‐Couto, Prado‐Medeiros, & Oliveira, 2014).

Furthermore, fibrosis is characterized by abnormal accumulation of extracellular matrix (ECM), and it can also interfere with function (Lieber & Ward, 2013). The transforming growth factor‐β (TGF‐β) cytokine has been considered as the master switch for the induction of fibrosis. The increase in TGF‐β1 expression is followed by increased collagen in denervated muscles (Zhang, Zheng, Chen, & Chen, 2008). In addition, myostatin, a member of the TGF‐β superfamily (Costamagna, Costelli, Sampaolesi, & Pennsa, 2015; Lieber & Ward, 2013; Snijders et al., 2015; Triandafilou & Kamper, 2012), is able to regulate skeletal muscle growth and to induce fibrosis.

The adaptations of skeletal muscle in upperlimb of humans with hemiparesis were also described. Muscle atrophy (Berenpas, Martens, Weerdesteyn, Geurts, & Alfen, 2017; Ploutz‐Snyder, Clark, Logan, & Turk, 2012), high stiffness, and increased amount of fibrous and fat tissues (Ryan, Dobrovolny, Smith, Silver, & Macko, 2012; Lee, Spear, & Rymer, 2015; Ploutz‐Snyder et al., 2012) were observed. Considering the focal cortical ischemia induced by endothelin‐1 was able to induce connective tissue proliferation in the endomysium and perimysium, muscle atrophy, and neuromuscular impairment in coordinated tasks of forelimb, the endothelin‐1 model seems to present a good translational interface. However, future studies should focus on molecular biomarkers to understand the skeletal muscle physiopathology in this model.

As limitations of this study, no identification of processes of stability proteins and motor‐plate regulation were performed, which could confirm the (morphometric) expansion of the neuromuscular junctions evaluated. Likewise, brain tissue analysis was not performed beyond the simple confirmation of induced cerebral ischemia. Molecular and neurophysiological analyses could also bring important insights into the present results.

In conclusion, focal brain ischemia‐induced NMJ expansion, muscle fiber atrophy, and fibrosis in muscles of paretic forelimb. These alterations are related to neuromuscular performance in well‐coordinated movements of forelimb. The present study brought important aspects for understanding compensatory mechanisms for function of forelimb.

## CONFLICT OF INTEREST

None declared.

## Data Availability

The data that support the findings of this study are available from the corresponding author upon reasonable request.
